# Religiosity, Emotions, Resilience, and Wellness during the COVID-19 Pandemic: A Study of Taiwanese University Students

**DOI:** 10.3390/ijerph18126381

**Published:** 2021-06-12

**Authors:** Inna Reddy Edara, Fides del Castillo, Gregory Siy Ching, Clarence Darro del Castillo

**Affiliations:** 1 Graduate Institute of Educational Leadership & Development, Fu Jen Catholic University, New Taipei City 24205, Taiwan; 065049@mail.fju.edu.tw; 2 Theology and Religious Education Department, De La Salle University, Manila 1004, Philippines; fides.delcastillo@dlsu.edu.ph; 3 Research and Development Center for Physical Education Health and Information Technology, Graduate Institute of Educational Leadership & Development, Fu Jen Catholic University, New Taipei City 24205, Taiwan; 4 Administration Office, Lumina Foundation for Integral Human Development, Calamba City 4027, Philippines; cdbdelcastillo@gmail.com

**Keywords:** religiosity, wellness, health, self-responsibility, emotions, resilience, pandemic

## Abstract

One hard fact of COVID-19 is the uncertainty of all things. Anchoring on the assumption that the religiosity of an individual has a profound impact on their emotions, resilience, and wellness, this study investigated the levels of the centrality of religiosity, emotions towards God, resilience, and wellness among 399 Taiwanese university students. Data analysis included descriptive statistics, factor analysis, group comparisons, multiple regression, and mediation analysis. Findings showed that most of the participants were religious. Furthermore, the 16 emotions towards God were successfully factored into three distinct sub-groups, namely: pleasant, unpleasant, and moral valence, which were later found to be quite related to Asian religions. More importantly, the results suggested that the resiliency of an individual can be attributed to their belief in the existence of God or the Divine, while the wellness indicators of security and satisfaction were related to one’s religiosity. Lastly, structural equation modeling showed that resilience fully mediated the relationship between the ideology dimension of religiosity and the security and satisfaction component of wellness. In addition to discussing these significant results, this paper also included some implications of the study results, particularly the importance of religiosity and emotions toward God or the Divine in sustaining resilience and promoting wellness, especially in the context of crisis, such as the current COVID-19 pandemic.

## 1. Introduction

The onset of the COVID-19 pandemic has created a world filled with uncertainties. In Taiwan, the COVID-19 pandemic has been handled with strict contact tracing and quarantine rules [1]. Although the use of technology has helped prevent the spread of COVID-19 [2], misinformation from the internet has also brought about many uncertainties of the pandemic [3]. Within an educational setting, the opening up of schools or conducting online classes has also been troubled with uncertainties [4]. Issues from the quality of teaching [5] to the medium of instructions [6] have together highlighted the impact of COVID-19 on education. In Taiwan, schools are successfully kept open with strict rules and restrictions [7]; nonetheless, the anxiety and psychological wellness of individuals are still being affected [8].

Considering ones’ internal belief systems and feelings, it could be said that human emotionality has been a constituent element of religiosity and that the feeling of one sort or another has been integral to society and religion [9]. The role of emotions in religiosity and the experience of the Divine has been a subject of various disciplines, mainly of theological and anthropological investigations [10]. In the recent past, this subject has also aroused a modest interest in the field of psychology, explicitly investigating that religion may serve as a source of certain emotions and may also lead to emotional health [11]. This also holds base within the context of the current COVID-19 pandemic, wherein religion, religiosity and emotions are essential factors in the coping process [12,13,14]. Thus, it can be considered common sense that religiosity, which includes some kind of religious practices and beliefs, is closely linked to people’s emotional experiences and wellness evaluations. Irrespective of religious affiliation, people across the spectrum of various geographic locations experience and express an array of emotions in their religious conduct, which is inseparable from a dogmatic or informal belief in God or the Divine.

### 1.1. The Link between Emotions, Religiosity, and the Divine

Although systematic research about emotions in the context of religiosity had not begun until the early years of this century [11], already, at the onset of the 20th century, James [15] identified the dimension of emotional rigor and its enthusiastic temper of espousals of the hallmark of religion for any religion to mean anything definite for the respective believers (p. 48). In elaborating the underlying reasons for such links between religiosity and a unique profile of emotional experiences, subsequent research evidence suggested that links between religiosity and emotional experiences reflect how the believers react emotionally and regulate emotions in the context of religious rituals and events [10,16,17,18,19].

The common denominator in the systematic definition of any religion is the belief in God or the Divine, regarded as either an immanent or transcendental Being [20]. As a type of attachment figure, recognizing a transcendental or immanent Being is a religious imperative for many believers who actively seek to be close to God or the Divine that they believe in [21], and to whom some elevated characteristics are attributed, such as being benevolent, omnipotent, and omniscient [22]. Fostering, maintaining, and developing such a belief in a supernatural attachment figure can be facilitated and regulated by emotions that are elicited in a religiosity context [19,23]. Furthermore, some even proposed that seeing God or the Divine figure as cruel or distant might be linked with feelings of anger, doubt, and fear of God’s disapproval [24]. For instance, the advent of the COVID-19 pandemic might be inferred to God as currently being angry [25].

Thus, emotions elicited in a religiosity context can foster and elevate a systematic belief in a deity. Haidt [26] distinguished between two categories of emotions as other-praising and self-praising. Other-praising emotions are elicited in response to the positive attributes of others and self-praising emotions are aroused in response to the positive attributes of oneself. Some suggested that belief in either transcendental or immanent supernatural beings may be facilitated by other-praising emotions and impaired by self-praising emotions [19]. For instance, in the context of religiosity, other-praising emotions may comprise awe and gratitude [17,26,27,28].

Furthermore, instances were found within the negative emotion of guilt in the context of religiosity among adolescents and young adults [29]. Similarly, COVID-19 induced negative emotions which are also said to be related to the feeling of mistrust in the Divine [30]. In essence, these explanations suggest that believers do experience emotions relative to their relationship with God or the Divine, or some sort of supernatural aspects. 

### 1.2. Sociocultural Underpinnings of Religiosity and Emotions toward the Divine

There is a substantive link between emotions, religiosity, and the Divine. However, it also should be acknowledged that emotions and religiosity are not exclusively located within a universal framework. Drawing on a range of research scholarship results, scholars charted the patterns of changes in emotionality over time, from place to place, and how its role in religiosity has varied accordingly. For example, fear of God meant something different to the 17th century Puritans than it did to the 20th century Evangelicals; key emotions associated with family relationships in the order of Confucian ideals in Korea had changed as the Buddhist religious ideas became prominent, and Indonesian Muslims interwove feelings with the recital of scriptures. Likewise, emotions that are central to the religious rituals in one community may not be important in other communities. For instance, the Newar community of Nepal experiences an emotion called nuga, which is a complex amalgamation of physical sensation, cognitive judgment, moral reasoning, and consciousness of the Divine [9].

Furthermore, Schaap-Jonker [23] pointed out that “one cannot study God representations without considering what culturally specific systems, as bearers of religious traditions, also constitute the content of these representations” (p. 12). Along with this point, Krause and Ironson [31] explored variations among Caucasians, African Americans, and Hispanics about their positive God-images and positive emotions toward God. Their results suggested African Americans were more likely than the other two ethnic groups to have strong positive emotions toward God. Thus, Corrigan [9] proposed to take the social scientific approaches in investigating the relationship between religiosity and emotion, for such approaches make it possible to observe how a certain emotion is aroused and expressed by persons in different religious contexts and various cultural settings.

### 1.3. Interrelationship of Religiosity, Emotions, Resilience, and Wellness

Studies have shown that both religiosity and resiliency played an important role in promoting wellness in times of pandemic [32,33]. For instance, in Portugal, healthcare and frontline workers have all noted the significant effects of religiosity in fostering resiliency [13,34]. Further, resilience has been shown to improve the subjective and psychological well-being of faculty during COVID-19 [35]. Concerning students during COVID-19, both stress and wellness have been a persisting issue of concern [36]. It has been reported that depression and the well-being of students were correlated with COVID-19 [37,38]; however, it was also noted that coping was better facilitated, as long as students were more resilient [39].

Moreover, although there exists an evidence of some studies in the religiosity of individuals, there is no evidence of assessment tools developed or empirical studies conducted regarding the emotions toward God or the Divine in Taiwan. Therefore, given the presence of a multi-religious context in Taiwan with the majority of the population adhering to some sort of religious practice, it appears to be very necessary to validate with the Taiwanese sample the already existing “Emotions toward God” inventory [40]. Moreover, the results from this empirical validation could be appropriately interpreted in the cultural and religious context of Taiwanese people’s emotions toward God or the Divine, and the validated inventory itself could be used in future empirical studies with the Taiwanese samples. Lastly, the relationship of religiosity, emotions, resilience, and wellness in times of the COVID-19 pandemic is also an area that needs to be explored.

Therefore, the current paper specifically addressed the following questions:

- What distinct emotions toward God are prevalent within the Taiwanese university student context?
- What are the levels of religiosity, resilience, and wellness within the Taiwanese university student context?
- What are the interrelationships that exist between the students’ religiosity, emotions, levels of resilience and wellness, with respect to their demographic background?
- What is the role of religiosity, emotions toward God, and resilience in the wellness of the participants?

## 2. Materials and Methods

**Figure 1:**
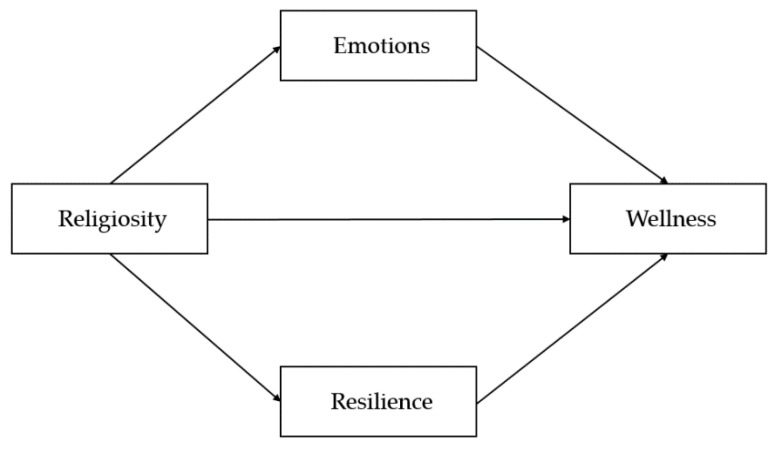
Conceptual framework of the study.

The current study was designed with a descriptive approach in mind, wherein a survey was used to collect the information best used in describing the phenomenon under survey [41]. In addition, the study also hypothesized that the students’ wellness would be highly affected by their religiosity, emotions, and resilience. Figure 1 shows the conceptual diagram of the study.

In general, religiosity can be noted as the sense of having a belief or religion [42], while emotions are the feelings felt about God or something Divine [40]. Furthermore, resilience can be said to be the capacity to adapt when faced with difficulties [43]. As these components are assumed to be interrelated, it was hypothesized that the students’ emotions and resilience would be regulated by the level of religiosity in fostering wellness. Conversely, they were all hypothesized to effect wellness, which was considered as the physical, social, mental, and emotional health of an individual [44].

### 2.1. Research Instruments

#### 2.1.1. Centrality of Religiosity (CRS)

To understand the religiosity of Taiwanese university students, the interreligious version of the CRS was used [42]. For countries that are primarily non-Christian, such as the case of Taiwan [45], the assessment of religiosity should therefore include a much broader concept [46]. For instance, the issue such as praying should be expanded to relate better to Asian societies, wherein the use of incense is equivalent to praying to or communicating with the Divine [47]. 

Huber and Huber [42] proposed that an individual’s religiosity can be represented by five main subscales or dimensions, such as Intellect with sample items of: “How often do you think about religious issues?” and “How interested are you in learning more about religious topics?” Ideology with sample items of: “To what extent do you believe that God or something divine exists?” and “In your opinion, how probable is it that a higher power really exists?” Public practice with sample items of: “How often do you take part in religious services?” and “How important is it for you to be connected to a religious community?” Private practice with sample items of: “How often do you pray?” and “How important is personal prayer for you?” Experience with sample items of: “How often do you experience situations in which you have the feeling that you are in one with all?” and “How often do you experience situations in which you have the feeling that God or something divine is present?” (p. 717). Being a highly-used scale to measure the level of religiosity, the CRS measures the intensity of its five main dimensions to depict a form of description of the frequency and strength of an individual’s religious system. For more information on the CRS, please refer to Huber and Huber [42].

More specifically, this study used the translated version of the interreligious CRS (CRSi) validated by del Castillo et al. [48]. Within their study, the 14-item CRSi was validated and considered as an appropriate scale in measuring religiosity within a Taiwanese university context. For the current study, Cronbach’s [49] alpha reliability of the CRSi-14 was computed at 0.87, while the reliabilities of the CRSi-14 subscales were computed to have alpha values ranging from 0.61 to 0.89, signifying adequate to sound internal consistencies [41]. Confirmatory factor analysis (CFA) of the CRSi-14 was also accomplished with results denoting a mediocre fit with Chi-Square value of 113.47 at *p* = 0.000 and degrees of freedom (*df*) = 25, root mean square error of approximation (RMSEA) = 0.09 with 90% confidence intervals of 0.08 to 0.11, standardized root mean square residual (SRMR) = 0.043, goodness of fit (GFI) = 0.95, Tucker–Lewis coefficient (TLI) = 0.91, and comparative fit index (CFI) = 0.95, all of which are within the acceptable limits [50,51].

#### 2.1.2. Emotions towards God (EtG)

In 2010, Huber and Richard developed the 16-item Inventory of Emotions towards God (EtG). The scale captures the specific emotional tendencies which a subject generally feels towards God. EtG has two orthogonal factors: positive and negative emotions. These factors represent the psychological valence of emotions towards God. Moreover, the scale operationalizes the intensity of emotions toward God according to the frequency of situations in which they are perceived [40]. They are operationalized, for instance, when people pray to God for the provision of their needs as they trust the Divine to supply them with their needs.

Künkler et al. [29] reported that the works of Petersen [52] and Murken [53] were milestones in the empirical research on emotions towards God. The 24-item questionnaire of Petersen [52] and the 27-item questionnaire of Murken [53] list the possible emotions a person may have towards God, and subjects rate how strongly they feel each emotion. Building on these works, Huber and Richard [40] proposed the EtG inventory that measures the positive and negative emotions towards God with fewer items. More importantly, the EtG differentiates the positive and negative emotions into meaningful components based on theological concepts relevant to religiosity of people.

The EtG consists of 16 emotions which individuals are most likely to experience in relation to their religiosity. These emotions are as follows (including the translation used in the current study): protection (保佑 bǎo yòu or 保護 bǎo hù), joy (喜悅 xǐ yuè, awe (敬畏 jìng wèi), gratitude (感謝 gǎn xiè), trust (相信 xiāng xìn), happiness (幸福 xìng fú), reverence (尊敬 zūn jìng), hope (希望 xī wàng), release from guilt (從罪惡感中釋放 cóng zuì è gǎn zhōng shì fang), fear (恐懼 kǒng jù), anxiety (焦慮 jiāo lù), failure (失敗 shī bài), guilt (罪惡 zuì è), shame (恥辱 chǐ rǔ or 愧疚 kuì jiù), anger (生氣 shēng qì), and rage (憤怒 fèn nù) [40] (p. 40). Data were collected using a 5-point Likert [54] type scale (never, rarely, occasionally, often, and very often) on the perceived frequency of experiencing different emotions. For the current study, Cronbach’s [49] alpha reliability of the EtG was computed at 0.94, which signifies good internal consistency.

#### 2.1.3. Brief Resilience Scale (BRS)

The current study used the 6-item Brief Resilience Scale (BRS), developed by Smith et al. [55]. BRS was created to measure the ability of an individual to bounce back or recover from stressful events [55] which is a highly-used scale whose sample items include: “I tend to bounce back quickly after hard times” and “I have a hard time making it through stressful events” (reverse scored) [55] (p. 196). Data were collected using a 5-point Likert [54] type scale (strongly disagree, disagree, neutral, agree, and strongly agree) on the perceived agreement with the different statements. Cronbach’s [49] alpha reliability of the BRS was computed at 0.82, which signifies an adequate internal consistency.

#### 2.1.4. Students’ Wellness Scale (SWS)

The 8-item Students’ Wellness Scale (SWS) is a self-made inventory, wherein issues such as health, diet, and exercise were included [56,57]. Sample items include: “I have enough daily exercise” and “I eat breakfast every day.” In addition, items with regards to students’ sense of responsibility towards their safety were also included [58], such as “I wear a helmet when riding a bicycle or scooter.” This is because scooter-related incidents are the highest amongst university students in Taiwan [59]. Lastly, items regarding the students’ perceived security and satisfaction were asked. Sample items included were: “I am happy with my school” and “I feel safe at my school.” Data were collected using a 5-point Likert [54] type scale (strongly disagree, disagree, neutral, agree, and strongly agree) on the perceived agreement with the different statements. Cronbach’s [49] alpha reliability of the SWS was computed at 0.68, which signifies mediocre internal consistency.

### 2.2. Participants and Procedure of the Study

The current study was conducted within a private university located in the northern part of Taiwan. Data were collected from November 2020 to January 2021 using the convenience sampling method, wherein volunteer participants were students of a Philosophy of Life course. The Philosophy of Life course is a required subject, wherein topics of discussions include (but are not limited to): knowing oneself, family and relationships, social justice, life and death, faith and religion, life appreciation, humanistic care, and many others. The survey was accomplished as part of the in-class teaching. Informed consent was provided and students were free to participate and withdraw from answering the survey questions without any consequences. A total of 450 surveys were disseminated. After removing invalid returns and discounting the non-participating students, a total of 399 participants or 88.67% responses had valid data, which were then encoded and analyzed with the use of the SPSS software version 20 (IBM, Armonk, NY, USA) at loan from the university.

Table 1 shows the demographic profile of the participants. In Taiwan, religious beliefs are diverse and multifaceted, which are freely adapted to the contextualized realities and, at the same time, coexist in harmony [60]. Reports have noted that about 81% of the Taiwanese population is affiliated with some kind of religion [61]. Some common religions are Buddhism, Taoism, Christianity (including Protestants and Catholics), Yiguandao, and Folk religions (or traditional religions) [62,63]. Hence, data collected for this study also reflected the diversity within the students’ religious affiliation.

Of the 399 university student participants, 228 or 57.14% were female students, while the remaining 171 or 42.86% were male students. The average age of the participants was 20.62 years old with the age of female and male students almost identical (female students’ mean age was 20.66, while male students’ mean age was 20.57 years old). Majority of the participants claimed that they were Atheists (*n* = 168 or 42.10%), which was followed by Asian religions such as Taoism (*n* = 95 or 23.80%), Buddhism (*n* = 46 or 11.50%), and Folk religions (*n* = 52 or 13.00%). The rest of the participants claimed to be Protestants (*n* = 27 or 6.80%) and Roman Catholics (*n* = 11 or 2.80%).

### 2.3. Data Analysis

The expression of emotions typically varies by culture [64] and sometimes even differs by gender [65]. Within Chinese society, the expression of emotions is highly regulated concerning group harmony and status hierarchies [66]. With having these said, the current study also hypothesized that the emotions related to God or the Divine should also be distinct for the Taiwanese youth. Hence, exploratory factor analysis (EFA) was used to analyze the interrelationships among different EtG emotions [50]. Furthermore, Structural Equation Modeling (SEM) was also used to further validate the relationships among the EtG emotions [51]. 

Descriptive statistics were computed together with the means, standard deviations (SD), measures of skewness and kurtosis, and correlations. Furthermore, independent sampled *t*-tests were also used to compare the various factors with respect to gender and religiosity, while one-way analysis of variance (ANOVA) was conducted to determine the relationship between different religions. Regression analysis was conducted to determine the predictability of various factors. Lastly, SEM was also used to determine the causal relationships between religiosity, emotions, resilience, and wellness.

## 3. Results and Discussion

### 3.1. Emotions towards God Prevalent Within the Taiwanese Youth

In this study, selected Taiwanese university students responded to the EtG scale. They were asked how often they experience situations where they feel specific emotions towards God. Within the EtG analysis, Huber and Richard [40] asserted that emotions are deeply rooted and have a strong connection to physiological processes compared to beliefs. As such, religious experiences (religiosity and the corresponding emotions towards God) have a more profound impact on psychological makeup than religious beliefs (p. 22). In addition, individuals who sincerely believe in God’s existence are more likely to experience positive and negative emotions towards God or the Divine.

To better understand different emotions that university students felt about God or something Divine, the factorability of the 16 EtG emotions was tested under several criteria for factor analysis. Table 2 shows the various inter-correlations of the EtG emotions. Kaiser–Meyer–Olkin (KMO) value was computed at 0.92, above the acceptable value of 0.50 [67], while Bartlett’s test of sphericity was computed to be at 5856.13 with significant Chi-square (*p* < 0.000) and a *df* of 120.

The principal component analysis with Varimax rotation was also conducted, while the eigenvalues were computed to be greater than 1 [68]. Three factors emerged, accounting for 76.50% of the total variance. Each of the factors or subscales of pleasant valence, unpleasant valence, and moral valence accounted for 36.01%, 28.21%, and 12.28% of the variance, respectively. Pleasant valence is mostly considered positive, while unpleasant valence are the emotions that constitute negative feelings. Moral valence are the emotions of fear and guilt. Table 3 shows the descriptive statistics of the EtG emotions, together with the communalities of the items with values greater than 0.40 and factor loadings greater than 0.60 [69]. Computation for the measures of skewness and kurtosis are also provided with various Cronbach’s [49] alpha reliabilities of the subscales, all of which are within the acceptable parameters [41]. 

Further validation was made with the use of Structural Equation Modeling (SEM). First, the data were assessed for their normality by analyzing their skewness and kurtosis and the results were found to be within the acceptable ranges [70,71]. Second, multivariate normality was computed with the use of Mardia’s [72] coefficients. Results of the computation have shown that the collected data violated the assumption of multivariate normality [73]. To remedy this situation, the bootstrapped method was adapted to the subsequent SEM analysis [74,75], which showed that the proposed three factors of EtG emotions had a mediocre fit with Chi-Square value of 486.43 at *p* = 0.000 and *df* = 91, RMSEA = 0.10 with 90% confidence intervals of 0.09 to 0.11, SRMR = 0.066, GFI = 0.87, TLI = 0.91, and CFI = 0.93, all of which are within the acceptable limits [50,51]. Hence, the proposed three factors (pleasant, unpleasant, and moral valences) of EtG emotions appeared to be an adequate model in describing the Taiwanese youth.

Note that within the 16 EtG emotions, the *sense of protection* in relation to religiosity is considered the highest with a mean of 3.14 (SD = 1.16), while the unpleasant emotion *shame* scored the lowest with a mean of 1.78 (SD = 0.85). These findings are quite interesting and require further studies.

### 3.2. The Meaning of Religiosity, Resilience, and Wellness within the Taiwanese Student Context

#### 3.2.1. Centrality of Religiosity (CRSi-14) and Emotions toward God (EtG)

Taiwan is known for its religious diversity [76]. Hence, in order to better understand the participants’ levels of religiosity, CRSi-14 was used. Results showed that the mean score for CRSi-14 was computed at 2.73 (SD = 0.59), signifying that the sample consisted of a group of individuals with average religiosity [42] (p. 720). Correlations among the EtG subscales, CRSi-14, and participants’ ages are provided in Table 4. In addition, discriminant validity of the EtG subscales was also assessed using the Fornell and Larcker [77] method by comparing the square root of each of the average variance extracted (AVE) (pleasant valence = 0.81, moral valence = 0.79, and unpleasant valence = 0.87) with the correlation coefficients for each of the EtG subscales.

Table 4 shows that the square root of AVE (values in bold) is higher than the correlations, hence, signifying that the measurement model supported the discriminant validity between the EtG subscales [77]. Furthermore, composite reliability (CR) was also computed with values higher than 0.70, while AVE was with values higher than 0.50, all within the recommended range [50]. In addition, the table also reveals that the EtG subscales were not correlated with the participants’ age and religiosity (CRSi). These findings are quite interesting; it would seem that emotions are somewhat *not* affected by the students’ religiosity or age. As for the correlations among the EtG subscales, results show that three subscales were quite correlated with each other. As for CRSi-14, findings show that religiosity was positively correlated with age with *r* = 0.12 at *p* < 0.05, denoting that as the students grow older, they tend to become more interested in religion, and at the same time, they experience higher religiosity.

To better understand different EtG emotions, cross-tabulations were accomplished with various groups of religious affiliation. For simplification of analysis, the religious affiliations were further regrouped into three distinct classifications: Asian for the typical East Asian religions such as Taoism, Buddhism, and folk religion; Christian for Roman Catholic and Protestant groups, and lastly, atheists for those participants who claimed no religious affiliation. Furthermore, ANOVA was also computed to determine if there were any significant differences between the EtG emotions and the three groups of religious affiliation.

Table 5 shows various breakdown results with the *sense of protection* as the highest emotional attachment with God or the Divine regardless of religious affiliation. Similarly, *shame* also scored the lowest across the three religious groups. Furthermore, ANOVA results showed some significant differences between the EtG emotions of the three groups of religious affiliation. Interestingly, significant differences were found in all of the unpleasant valence emotions with the inclusion of fear (moral valence), wherein students with Asian religions experienced significant higher emotions in relation to God or something Divine more than their atheist counterparts, for instance: rage [F (2, 396) = 4.16, *p* = 0.016], anger [F (2, 396) = 4.56, *p* = 0.011], shame [F (2, 396) = 3.75, *p* = 0.024], Failure [F (2, 396) = 4.18, *p* = 0.016], and anxiety [F (2, 396) = 4.11, *p* = 0.017]. Similarly, fear [F (2, 396) = 4.66, *p* = 0.010] was also found to have significantly higher values for participants with Asian religious affiliations. These findings would suggest that although there were no distinct differences between religious affiliations and pleasant (positive emotions) valence, there were somehow significant differences when it comes to unpleasant (negative emotions) valence and the emotion of fear. Research suggested that emotions are correlated with the level of religiosity [78], while East Asian religions might be more accommodating [79]. Nonetheless, there is no definitive proof that atheists are less sensitive to emotions than the people with particular religious affiliations [80,81]. Additionally, note that some of the mean scores on EtG subscales and corresponding emotions of the participants who claimed to be atheists were sometimes even higher than the students who claimed to be Christians. However, this cannot be justified since no significant statistical meanings were found between the mean values. 

Significant differences were also found between the CRSi subscales and three groups of religious affiliation. Table 6 shows various mean scores of the subscales, wherein ANOVA results showed that participants with Asian and Christian religious affiliations scored significantly higher than their atheist counterparts. This finding is in line with the previous findings of del Castillo et al. [48] (p. 11). In addition, independent sampled *t*-tests were also conducted to determine if there were gender differences within the EtG emotions and CRSi subscales. Findings showed no significant gender differences, suggesting that for the Taiwanese university students, religiosity is not gender-specific. Lastly, an additional comparison was made between the emotions of religious and highly religious individuals (classification based on Huber and Huber [42]). Huber and Richard’s [40] original study proposed that emotions should be more profound with highly religious individuals. However, in the current study, the independent sampled *t*-test showed no significant differences in terms of EtG emotions between the religious and highly religious students. 

#### 3.2.2. Resilience (BRS)

As for the students’ level of resilience, BRS findings suggest that the Taiwanese university students were moderately resilient with a mean score of 3.10. Table 7 further shows the various BRS mean scores for three groups of religious affiliations with ANOVA resulting in no significant differences. Similarly, no significant gender differences were found. 

#### 3.2.3. Wellness (SWS)

As noted earlier, the SWS scale was a self-made instrument. Hence, it would also be logical to validate the factorability of the items. Table 8 shows various inter-correlations of the SWS items. KMO value was computed at 0.72, which is above the acceptable value of 0.50, while Bartlett’s test of sphericity was computed to be at 454.51 with significant Chi-square (*p* < 0.000) and a *df* of 28.

The principal component analysis with Varimax rotation was also conducted with three factors accounting for 59.29% of the total variance. Each of the factors or subscales of health and diet, safety and responsibility, and security and satisfaction accounted for 24.71%, 19.10%, and 15.48% of the variance, respectively. Table 9 shows the descriptive statistics of the SWS subscales and items, together with the communalities of the items with values greater than 0.40 and factor loadings greater than 0.60. Computation for the measures of skewness and kurtosis are also provided with the various alpha reliabilities of the subscales. Note that the alpha reliability for the subscale safety and responsibility with only two items was quite low with 0.35, denoting problematic internal consistency. However, this result would still be valid for further examination and analyses [82].

Succeeding SEM computations showed that the proposed three factors of SWS had an adequate fit with Chi-Square value of 45.95 at *p* = 0.000 and *df* = 17, RMSEA = 0.065 with 90% confidence intervals of 0.04 to 0.09, SRMR = 0.042, GFI = 0.97, TLI = 0.89, and CFI = 0.93—all of which are within the acceptable parameters. Therefore, SWS can be considered a reliable instrument except for the subscale safety and responsibility, which needs further testing and validation.

Furthermore, Table 10 shows various SWS mean scores for three groups of religious affiliations, denoting moderate mean scores. Similar to BRS, the ANOVA results showed that there were no significant differences between SWS and religious affiliations. In addition, there were no significant gender differences.

### 3.3. Interrelationships between CRSi, EtG, resilience, and wellness

As noted earlier, another focus of the current study was to determine the role of religiosity and emotions toward God or the Divine in the resilience and wellness of students. Therefore, to understand and predict resilience, step-wise multiple regression was performed. Results showed that only one CRSi subscale of ideology (*β* = 0.10, *p* = 0.020) significantly explained 1.4% of the variance in resilience with *F* (1, 397) = 5.46, *p* = 0.020), and an overall model fit of R^2^ = 0.014 and adjusted R^2^ = 0.011. This result suggested that the resiliency of an individual can be attributed to their higher levels of religiosity.

Similarly, several step-wise regressions were also performed to determine the predictors of wellness (together with its subscales). Findings showed that only the SWS subscale of security and satisfaction resulted in a significant model fit. Step-wise multiple regression showed that only one CRSi subscale of intellect (β = −0.13, *p* = 0.020) significantly explained 1.4% of the variance in the students’ sense of security and satisfaction, with *F* (1, 397) = 5.48, *p* = 0.020, and an overall model fit of R^2^ = 0.014 and adjusted R^2^ = 0.011. The negative regression weight denotes that low levels of religiosity as assessed by intellect leads to lower levels of security and satisfaction.

In addition, as noted with the previous analyses that some EtG emotions are sensitive to religious affiliation, several step-wise multiple regressions were also performed. Findings showed that all three EtG subscales (pleasant, unpleasant, and moral valences) were successfully predicted by Asian religions. Table 11 shows various regression coefficients and model fits.

**Figure 2:**
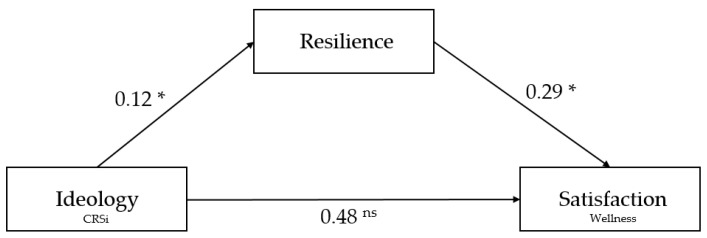
Standardized regression coefficients for the relationship between ideology and security and satisfaction mediated by resilience.* *p* < 0.05, ^ns^ non-significant, ^Wellness^ Students’ Wellness Scale, ^CRSi^ Interreligious Centrality of Religiosity Scale.

Lastly, several models were tested to determine the relationship between CRSi, EtG, resilience, and wellness. More specifically, it was hypothesized that the students’ wellness would be affected by their religiosity, emotions toward God, and resilience. Anchoring on the previous regression results, this hypothesized model was tested using SEM (as depicted in Figure 2).

SEM results showed that the relationship between the CRSi subscale—ideology and SWS subscale—security and satisfaction was fully mediated by *resilience*. As Figure 2 shows, the standardized regression coefficient between ideology and resilience was statistically significant (0.12, *p* < 0.05), as was the standardized regression coefficient between resilience and satisfaction (0.29, *p* < 0.05). The standardized total indirect effect was computed with bootstrapping (applied 2000 times), which resulted in 0.03 at *p* < 0.05. More importantly, the total standardized direct effect between ideology and satisfaction was not significant (0.48, ns); hence, denoting full mediation. Results also indicated an adequate fit with Chi-Square value of 141.59 at *p* = 0.000 and *df* = 39, RMSEA = 0.081 with 90% confidence intervals of 0.07 to 0.10, SRMR = 0.057, GFI = 0.94, TLI = 0.91, and CFI = 0.94—all of which are within the acceptable parameters.

## 4. Conclusions and Implications

The COVID-19 pandemic continues to create havoc in almost all parts of the world, and has become an unprecedented challenge to public health, mental well-being, the economy, social and religious life, and the world of work and education. The pandemic brought about an awareness of existential threat to the fore, in terms of livelihood, personal relations, and the meaning of life. The COVID-19 pandemic impacted religion in various ways, including the cancellation of in-person religious services. It has also aggravated mental health issues, reflecting the widespread effects of health-related fears, as well as uncertainty, and even meaninglessness. Under these circumstances, this research project attempted to address the connections between religious affiliation, religiosity, emotions toward God or the Divine, resilience, and wellness. In particular, using the Taiwanese student sample, the research tried to investigate the effect of religiosity on the wellness of the participants through the affective component of emotions and the cognitive component of resilience.

Based on the findings of this study, the expression of emotions towards God or the Divine is distinct for the Taiwanese youth. For the select university students, the emotion of protection concerning God is considered the highest. In the pleasant valence, the participants from non-religious, religious, and highly religious groups achieved a high mean score on the emotional sense of protection. Embedded in their religious belief is the deep trust that the Divine offers protection and service to the people [83]. This might also hold true in the current situation of the COVID-19 pandemic. Katz [84] asserts that deities are believed to prevent different sicknesses as well as all manner of calamities. One example is the town of Donggang in one of Taiwan’s oldest fishing ports where frequent calamities have threatened the life of the local people. The people in Donggang have actively engaged in the worship of such deities as they deeply believe that the Divine can intercede and provide them with great protection. This belief that the invisible divine spirits provide protection has been submerged in the Taiwanese culture and implanted in the emotions of the believers.

The emotions of trust and hope also emerged as prevalent among the study participants. Life in general is not predictable. People experience ups and downs all along the way. Especially, in times of uncertainty, such as the current status brought about by the pandemic—when life throws a curveball, when everything feels shaky and incomprehensible, and when one cannot foresee an easy resolution—the emotions of trust and hope in God or the Divine become prominent. In the midst of devastating experiences, people feel the necessity to trust God and have hope that the testing times will pass over.

The other distinct unpleasant emotions toward God that are prevalent within Taiwanese university students are fear and anxiety. Fear and anxiety are frequently used interchangeably. However, in some literature, these emotions are different in terms of their manifestation and functions [85,86]. Fear occurs when the person experiences proximate and imminent danger, while anxiety is typified by tension and worry [87]. It makes sense in the context of crisis that fear and anxiety go together. Tension and worry seemed to engulf the whole world in the midst of the devastating COVID-19 pandemic, while fear seems to be so natural as the pandemic endangers life itself. Additionally, fear of God or the Divine has a purifying effect. In the case of the Taiwanese religiosity context and the emotions toward God, fear was experienced periodically. In the postwar period, temples were remodeled into Shinto shrines. The believers greatly feared the anger of the deities and braced themselves from natural calamities and plagues [88]. However, this emotion of fear moved the believers away from the wrath of the deities and helped them protect themselves in responding to a crisis. Hence, fear is considered as a moral valence.

Looking at the centrality of religiosity among Taiwanese youth, the results suggested that the participants were moderately high in their religiosity. The measure of religiosity used in this study is represented by five dimensions of intellect, ideology, public practice, private practice, and experience. Although close to half of the participants claimed to be atheists, the above average scores on the ideology component of religiosity suggest that most of the Taiwanese youth do believe in the existence of God or something Divine. In fact, the results also indicated that as the people age, their religiosity increases. The dimension of intellect indicates that the Taiwanese youth not only believe in the existence of God or the Divine but also frequently think of and are interested in learning religious issues and topics. The higher-level religiosity of the Taiwanese youth is also often experienced and expressed both privately and publicly.

Further, looking at the Taiwanese students’ understanding of emotions toward God across different religious groups, the study suggested that while East Asian religious traditions might be more devout, there is no definite proof that atheists have less sensitivity to emotions than those affiliated to some religious traditions. The study showed that in some instances, students who have no religious affiliation (atheists) tend to have higher emotions than the students who are Christians.

Resilience is the ability of an individual to bounce back or recover from stressful events. The results in this study suggested that the Taiwanese university students are moderately resilient across all age groups and religious denominations. While facing concerns, the participants seem to have an ability to withstand the crisis and look ahead for a new normal. It also shows their capacity to constructively respond to a crisis and operate with the given resources to build resilient environments to create alternative sources of well-being.

The wellness construct in this study included the issues pertaining to health, diet, exercise, sleep, responsibility towards safety, and feeling of security and satisfaction. The total wellness score of study participants was above the average, suggesting that the university students in Taiwan generally eat healthy, exercise regularly, sleep well, are responsible towards safety, and also feel safe, secure, happy, and satisfied. Even though it is acknowledged that sometimes college students let their health fall by the wayside due to their bad habits and the pressure of academics, the participants across all the religious and age groups in this study appeared to have maintained overall physical and mental wellness. 

In addition to assessing the prevalence of religiosity, emotions toward God, resilience, and wellness among the Taiwanese university students, this study also investigated the role of religiosity and emotions toward God in the resilience and wellness of the participants by using step-wise multiple regression analyses. In the analysis predicting resilience, only the ideology subscale of CRSi (religiosity) explained some significant variance, suggesting that resiliency in people could be attributed to their belief in the existence of God or some divine reality. In other words, having religiosity in general and a belief in God facilitates the development of a resilient functioning in the situations of adversity.

Similarly, after conducting several step-wise regressions to determine the impact of religiosity on wellness, only the subscale intellect of CRSi (religiosity) explained some significant variance in the security and satisfaction component of wellness. The intellect dimension of religiosity describes people’s thoughts about religious issues and their interest in learning religious topics. Therefore, it could be said that the intellect dimension of religiosity is a cognitive component. Additionally, the security and satisfaction component of wellness pertains to cognition through which a subjective evaluation of life is conducted. Therefore, it appears natural that the people’s thoughts about religious issues and topics influence the subjective evaluation of their lives in terms of security and satisfaction.

Lastly, this study pointed out that the Taiwanese students’ religiosity and its relation to wellness was regulated by their emotions toward God and resilience. The results indicated that only the ideology subscale of religiosity, where it is believed that some sort of the Divine exists, relates to the wellness dimension of security and satisfaction through resilience. Studies affirm that religiosity is associated with resilience and wellness [89,90,91]. During the COVID-19 pandemic, people seem to rely on the power of the Divine for intercession and interventions. The belief in the existence of God or the Divine signifies pleasant valence that leads to the resiliency of the person amidst the challenges of the crisis, such as the current pandemic. 

Therefore, as a conclusion, we can say that the study results affirm the prevalence of various levels of religiosity, irrespective of age groups or religious affiliation. Moreover, levels in religiosity and the emotions toward God do differ based on socio-cultural backgrounds and existing conditions. Additionally, resilience is activated in the midst of dealing with life challenges and it is backed by some dimensions of religiosity. Finally, religiosity, together with the ability of resiliency, promote overall wellness. Hence, assessing these dimensions in people, particularly when facing adversaries in life and intervening appropriately, are important for the promotion of their overall health and wellness. Particularly, acknowledging people’s religiosity and creating arenas for expressing and experiencing their religiosity in its various dimensions is worthy of consideration in building resilience and fostering wellness.

## 5. Limitations and Future Directions

Although this research study indicated some significant results, thus making a substantial contribution to the research and practice in the areas of religiosity, emotions, resilience, and wellness, it should be acknowledged that this study is not without limitations. The primary limitation is that the sample in this study was college students with a minimum age range and who tend to show less interest in religion or religiosity issues. Therefore, it is necessary to include various age groups and diverse samples to replicate the results from this study, and even to further advance the research in the fields of religion and wellness, particularly in the context of adversity. Second, some of the study variables probably were not appropriately conceptualized due to limited and self-created items, or due to translation and back-translation issues. Although these items and measures had acceptable inter-item correlations and reliability coefficients, it is still warranted to replicate these results with more and diverse populations. Finally, even though some advanced statistical analyses, such as SEM, were conducted in this study for obtaining valid results, it is still not justified to draw causal relations among the study variables. Future studies involving these same constructs should try to build coherent models while taking into consideration the population under study in any given situation. 

## Figures and Tables

**Table 1:** Demographic profile of the participants.

Demographics	Classification	*n*	*%*
Age ^1^	20	252	63
	21	82	21
	22	29	7
	23	36	9
	Total	399	100
Gender	Female	228	57
	Male	171	43
	Total	399	100
Religion	Taoism	95	24
	Buddhism	46	12
	Roman Catholic	11	3
	Protestant	27	7
	Traditional (Folk Religion)	52	13
	Atheist	168	42
	Total	399	100

Note: *N* = 399. ^1^ Age is in years. Mean age = 20.62.

**Table 2:** Correlation matrix of the EtG emotions.

Emotions	1	2	3	4	5	6	7	8	9	10	11	12	13	14	15
1. Protection															
2. Joy	0.50 **														
3. Awe	0.71 **	0.59 **													
4. Gratitude	0.60 **	0.77 **	0.65 **												
5. Trust	0.66 **	0.71 **	0.68 **	0.78 **											
6. Happiness	0.47 **	0.82 **	0.53 **	0.74 **	0.72 **										
7. Reverence	0.63 **	0.60 **	0.72 **	0.69 **	0.70 **	0.61 **									
8. Hope	0.63 **	0.65 **	0.63 **	0.73 **	0.75 **	0.72 **	0.68 **								
9. Release from Guilt	0.46 **	0.69 **	0.53 **	0.63 **	0.63 **	0.67 **	0.53 **	0.64 **							
10. Fear	0.41 **	0.37 **	0.44 **	0.37 **	0.43 **	0.37 **	0.38 **	0.43 **	0.37 **						
11. Guilt	0.37 **	0.34 **	0.41 **	0.36 **	0.40 **	0.39 **	0.33 **	0.41 **	0.40 **	0.62 **					
12. Rage	0.21 **	0.51 **	0.30 **	0.41 **	0.39 **	0.47 **	0.30 **	0.36 **	0.51 **	0.46 **	0.50 **				
13. Anger	0.23 **	0.52 **	0.32 **	0.43 **	0.42 **	0.49 **	0.33 **	0.37 **	0.51 **	0.45 **	0.51 **	0.95 **			
14. Shame	0.25 **	0.48 **	0.34 **	0.37 **	0.38 **	0.44 **	0.30 **	0.36 **	0.51 **	0.50 **	0.65 **	0.78 **	0.77 **		
15. Failure	0.23 **	0.45 **	0.32 **	0.39 **	0.37 **	0.46 **	0.30 **	0.37 **	0.44 **	0.54 **	0.58 **	0.70 **	0.71 **	0.71 **	
16. Anxiety	0.35 **	0.49 **	0.42 **	0.44 **	0.42 **	0.51 **	0.41 **	0.43 **	0.50 **	0.66 **	0.57 **	0.64 **	0.65 **	0.64 **	0.76 **

Note: ** *p* < 0.01.

**Table 3:** Descriptive statistics, communalities, factor loadings, and alpha reliabilities of EtG emotions.

EtG Subscales and Emotions	Mean	SD	Communalities	FL	Skewness	Kurtosis	Alpha
Pleasant Valence	2.76	0.95			−0.02	−0.33	0.94
Protection ^1^	3.14	1.16	0.74	0.69	−0.15	−0.60	
Joy	2.45	1.11	0.81	0.80	0.49	−0.37	
Awe	2.89	1.14	0.74	0.72	0.07	−0.56	
Gratitude	2.83	1.14	0.80	0.86	0.11	−0.58	
Trust	2.84	1.18	0.79	0.84	0.13	−0.68	
Happiness	2.54	1.16	0.79	0.79	0.47	−0.48	
Reverence	2.98	1.21	0.71	0.79	−0.03	−0.76	
Hope	2.94	1.19	0.75	0.81	0.04	−0.76	
Release from Guilt	2.25	1.03	0.66	0.68	0.63	−0.06	
Unpleasant Valence	1.94	0.82			0.66	0.05	0.93
Anxiety ^1^	2.15	1.04	0.73	0.68	0.75	0.05	
Failure	2.03	0.99	0.76	0.80	0.76	0.05	
Shame ^2^	1.78	0.85	0.79	0.83	0.93	0.58	
Anger	1.87	0.89	0.86	0.89	0.86	0.46	
Rage	1.85	0.88	0.86	0.90	0.87	0.41	
Moral Valence	2.25	0.97			0.48	−0.31	0.77
Fear ^1^	2.36	1.10	0.75	0.73	0.54	−0.32	
Guilt	2.14	1.06	0.71	0.64	0.67	−0.26	
Overall EtG	2.32	0.78			0.15	−0.74	0.94

Note: SD = standard deviation and FL = factor loading. Extraction method: principal component analysis. Rotation method: Varimax with Kaiser normalization. Rotation converged in 9 iterations. ^1^ Emotion with the highest score within each of the EtG subscale. ^2^ Emotion with the lowest score among the 16 emotions.

**Table 4:** Discriminant validity and correlation matrix for participants’ age, EtG subscales, and overall CRSi-14.

Factors	CR	AVE	1	2	3	4
1. Age						
2. Pleasant Valence	0.95	0.66	0.03	**0.81**		
3. Moral Valence	0.77	0.62	0.01	0.52 **	**0.79**	
4. Unpleasant Valence	0.94	0.77	0.09	0.54 **	0.69 **	**0.87**
5. Overall CRSi-14			0.11 *	0.01	0.01	0.04

Note: * *p* < 0.05 and ** *p* < 0.01. Values in bold are the square root of each of the average variance extracted.

**Table 5:** Cross-tabulation between EtG subscales and religious affiliation.

EtG Subscales and Emotions	Asian		Christian		Atheist		Total	
	Mean	SD	Mean	SD	Mean	SD	Mean	SD
Pleasant Valence	2.88	0.95	2.54	0.82	2.68	0.97	2.76	0.95
Protection	3.22	1.11	2.89	1.16	3.10	1.23	3.14	1.16
Joy	2.56	1.14	2.13	0.91	2.38	1.11	2.45	1.11
Awe	3.01	1.14	2.87	0.99	2.76	1.17	2.89	1.14
Gratitude	2.92	1.11	2.61	1.10	2.77	1.16	2.83	1.14
Trust	2.94	1.17	2.66	1.10	2.78	1.22	2.84	1.18
Happiness	2.66	1.18	2.29	0.90	2.46	1.17	2.54	1.16
Reverence	3.10	1.16	2.76	1.22	2.89	1.25	2.98	1.21
Hope	3.10	1.21	2.66	1.07	2.83	1.18	2.94	1.19
Release from Guilt	2.37	1.07	1.97	0.89	2.16	1.01	2.25	1.03
Unpleasant Valence	2.07	0.88	1.86	0.81	1.80	0.73	1.94	0.82
Anxiety	2.30	1.07	1.92	1.02	2.02	0.99	2.15	1.04
Failure	2.18	1.07	1.87	0.96	1.90	0.87	2.03	0.99
Shame	1.90	0.93	1.74	0.80	1.66	0.74	1.78	0.85
Anger	2.01	0.97	1.84	0.92	1.73	0.76	1.87	0.89
Rage	1.97	0.95	1.92	0.97	1.71	0.74	1.85	0.88
Moral Valence	2.39	0.98	2.16	0.98	2.10	0.94	2.25	0.97
Fear	2.52	1.12	2.32	1.21	2.17	1.03	2.36	1.10
Guilt	2.25	1.09	2.00	0.96	2.04	1.04	2.14	1.06
Overall EtG	2.44	0.82	2.18	0.71	2.20	0.72	2.32	0.78

Note: *N* = 399, Asian = 193, Christians = 38, and Atheist = 168. SD = standard deviation.

**Table 6:** Cross-tabulation between CRSi-14 subscales and religious affiliation.

CRSi Subscales	Asian		Christian		Atheist		Total	
	Mean	SD	Mean	SD	Mean	SD	Mean	SD
Intellect	2.80	0.76	3.25	0.96	2.48	0.70	2.71	0.79
Ideology	3.73	0.71	3.84	0.64	3.23	0.84	3.53	0.80
Public practice	2.34	0.63	3.09	0.77	1.97	0.51	2.26	0.68
Private practice	2.89	0.63	3.04	0.60	2.50	0.52	2.74	0.62
Experience	2.54	0.89	3.30	1.06	2.13	0.80	2.44	0.93
Overall CRSi-14	2.86	0.54	3.31	0.62	2.46	0.49	2.73	0.59

Note: *N* = 399, Asian = 193, Christians = 38, and Atheist = 168. SD = standard deviation.

**Table 7:** Cross-tabulation between BRS and religious affiliation.

Factor	Asian		Christian		Atheist		Total	
	Mean	SD	Mean	SD	Mean	SD	Mean	SD
BRS	3.09	0.69	3.26	0.70	3.07	0.58	3.10	0.65

Note: *N* = 399, Asian = 193, Christians = 38, and Atheist = 168. SD = standard deviation.

**Table 8:** Correlation matrix of the SWS items.

Wellness Items	1	2	3	4	5	6	7
1. I eat breakfast every day							
2. Fruits and vegetables are an important part of my diet	0.34 **						
3. I have enough daily exercise	0.28 **	0.33 **					
4. I have enough sleep every day	0.27 **	0.28 **	0.48 **				
5. I wear a seat belt when riding a car or bus	0.19 **	0.07	0.09	0.16 **			
6. I wear a helmet when riding a bicycle or scooter	0.14 **	0.25 **	0.24 **	0.16 **	0.21 **		
7. I am happy with my school	0.14 **	0.14 **	0.21 **	0.18 **	0.19 **	0.20 **	
8. I feel safe at my school	0.12 *	0.13 **	0.24 **	0.27 **	0.17 **	0.07	0.46 **

Note: ** *p* < 0.01 and * *p* < 0.05.

**Table 9:** Descriptive statistics, communalities, factor loadings, and alpha reliabilities of SWS.

Wellness Subscales and Items	Mean	SD	Com	FL	Skew	Kur	Alpha
Health and Diet	3.06	0.77			−0.05	0.23	0.66
1. I eat breakfast every day	3.35	1.17	0.43	0.69	−0.29	−0.69	
2. Fruits and vegetables are an important part of my diet	3.33	1.08	0.53	0.80	−0.31	−0.42	
3. I have enough daily exercise	2.76	1.06	0.61	0.72	0.20	−0.42	
4. I have enough sleep every day	2.79	1.08	0.56	0.86	0.14	−0.57	
Safety and Responsibility	2.74	0.93			0.09	−0.15	0.35 ^1^
1. I wear a seat belt when riding a car or bus	2.56	1.26	0.64	0.84	0.46	−0.78	
2. I wear a helmet when riding a bicycle or scooter	2.93	1.12	0.56	0.79	0.02	−0.56	
Security and Satisfaction	3.27	0.91			−0.23	0.26	0.63
1. I am happy with my school	3.27	0.91	0.67	0.79	−0.23	0.26	
2. I feel safe at my school	3.56	0.89	0.75	0.81	−0.19	0.00	
Overall Wellness	3.02	0.62			0.02	0.36	0.68

Note: SD = standard deviation, Com = communalities, FL = factor loading, Skew = skewness, and Kur = kurtosis. Extraction method: principal component analysis. Rotation method: Varimax with Kaiser normalization. Rotation converged in 5 iterations. ^1^ Alpha value below the accepted parameters.

**Table 10:** Cross-tabulation between SWS subscales and religious affiliation.

SWS Subscales	Asian		Christian		Atheist		Total	
	Mean	SD	Mean	SD	Mean	SD	Mean	SD
Health and Diet	3.10	0.77	3.19	0.72	2.98	0.78	3.06	0.77
Safety and Responsibility	2.72	0.91	2.80	0.91	2.76	0.96	2.74	0.93
Security and Satisfaction	3.30	0.90	3.11	0.95	3.28	0.93	3.27	0.91
Overall SWS	3.04	0.60	3.03	0.53	3.01	0.66	3.02	0.62

Note: *N* = 399, Asian = 193, Christians = 38, and Atheist = 168.

**Table 11:** Regression coefficients and models predicting EtG subscales by Asian religions.

EtG Subscales	*β*	*p*	*F*	*p*	R^2^	R^2^ Adjusted	% Variance Explained
Pleasant	0.22	0.020	5.50	0.020	0.014	0.011	1.40
Unpleasant	0.26	0.002	9.94	0.002	0.024	0.022	2.40
Moral	0.28	0.005	8.09	0.005	0.020	0.018	2.00

Note: df = 1, 397. *β* = regression coefficient. *F* = f statistics is the ratio of the mean regression sum of squares divided by the mean error sum of squares. R^2^ and R^2^ adjusted are the coefficient of determination.

## Data Availability

Data used in this study is available at https://doi.org/10.6084/m9.figshare.14445234.v2 (accessed on 20 April 2021).
